# Value of the clinical pharmacist interventions in the application of the American College of Cardiology (ACC/AHA) 2018 guideline for cholesterol management

**DOI:** 10.1371/journal.pone.0283369

**Published:** 2023-03-27

**Authors:** Mohammad M. AlAhmad, Sham ZainAlAbdin, Khozama AlAhmad, Iqbal AlAhmad, Salah AbuRuz

**Affiliations:** 1 Department of Clinical Pharmacy, College of Pharmacy, Al Ain University, Al Ain, United Arab Emirates; 2 AAU Health and Biomedical Research Center, Al Ain University, Abu Dhabi, United Arab Emirates; 3 Department of Pharmacology and Therapeutics, College of Medicine and Health Sciences, United Arab Emirates University, Al Ain, United Arab Emirates; 4 Mediclinic AlAin Hospital, Al Ain, United Arab Emirates; 5 Czech Rehabilitation Hospital, Al Ain, United Arab Emirates; 6 Department of Biopharmaceutics and Clinical Pharmacy, School of Pharmacy, University of Jordan, Amman, Jordan; Rinku General Medical Center: Rinku Sogo Iryo Center, JAPAN

## Abstract

**Objectives:**

The study aims to examine the extent to which the updated ACC/AHA management of blood cholesterol guideline (2018) is implemented in practice and to assess the value of the clinical pharmacist interventions in improving physicians’ adherence the guidelines recommendations.

**Methods:**

We utilized in this study an interventional before-after design. The study was conducted on 272 adult patients who visited the study site internal medicine clinics and were candidates for statin therapy based on the 2018 ACC/AHA guidelines for cholesterol management. Adherence to guideline recommendations was measured before and after clinical pharmacists’ interventions by calculating the percentage of patients receiving statin therapy as per guideline recommendation, the type and intensity (moderate or high intensity) of statin therapy used, and the need for additional non-statin therapy.

**Results:**

Adherence with guideline recommendations was significantly improved from 60.3% to 92.6% (X2 = 79.1, p = 0.0001) after clinical pharmacist interventions. Among patients who were on statin therapy, the percentage of those who were on proper statin intensity increased significantly from 47.6% to 94.4% (X2 = 72.5, p = 0.0001). The combination of statins with non-statin therapies such as ezetimibe and PCSK9 inhibitors increased from 8.5% to 30.6% (X2 = 95, p<0.0001) and from 0.0% to 1.6% (X2 = 6, p = 0.014), respectively. The use of other lipid-lowering agents was diminished from 14.6% to 3.2% (X2 = 19.2, p<0.0001).

**Conclusion:**

Collaboration between physicians and clinical pharmacists is a crucial strategy to improve patients’ treatment and hence, achieve better health outcomes among patients suffering from dyslipidemia.

## Introduction

Coronary heart disease (CHD) is considered one of the main causes of morbidity and mortality worldwide. One of the major risk factors that contributes to the development and progression of CHD is dyslipidemia [1]. According to the World Health Organization (WHO), an estimated 17.9 million patients died each year because of cardiovascular diseases, of which more than 80.0% of the deaths were due to stroke and myocardial infarction [2].

Ischemic heart diseases and stroke are among the top 3 leading causes of years of life lost (YLL) and mortality according to a global burden disease study [3]. Another recent study in UAE showed that the overall prevalence of dyslipidemia among adults was 72.5%, where the total cholesterol and LDL-C levels were high in 42.8% and 38.6% of the participants, respectively [4]. In addition, a study including an expatriate population in the UAE reported a high prevalence of either overweight or obesity (75.3%) as well as known associated risk factors for developing both metabolic syndrome and dyslipidemia [5].

A large number of clinical trials have reported the benefits of lowering cholesterol levels, particularly LDL-C, in reducing the mortality rate among CHD patients. Based on that, the American College of Cardiology (ACC/AHA) published the 2013 blood cholesterol treatment guidelines to reduce atherosclerotic cardiovascular risk in adults. This guideline has been updated several times since then [6]. The latest update of the ACC/AHA management of blood cholesterol guideline (2018) emphasizes on the importance of categorizing patients into the four statins benefit groups and on the importance of statin therapy using evidence-based intensity level (high- or moderate-intensity statins). Furthermore, it highlights the importance of adding PCSK9 inhibitor therapy after receiving the maximum tolerated statin therapy and ezetimibe to achieve LDL-C < 70 mg/dl or non-HDL-C < 100 mg/dl for some patients [7, 8].

The prevalence and treatment rates of dyslipidemia are high in the UAE and worldwide [4], however, it was found that a significant percentage of patients worldwide were not taking appropriate lipid-lowering agents or were taking statins but were not meeting the primary treatment goal [9, 10]. One of the reasons identified was the low adherence to the guideline recommendations. Clinical pharmacists play important role in individualizing patient treatment and improving adherence to guideline’s recommendations. The present study aims to examine the extent to which the updated ACC/AHA management of blood cholesterol guideline (2018) is implemented in practice and to assess the value of the clinical pharmacists’ interventions in improving physicians’ adherence to the guideline’s recommendations. In UAE, the ACC / AHA guidelines are the most commonly followed and recommended guidelines by the health authorities.

## Methods

### Subjects and settings

The study was conducted on adult patients attending an internal medicine clinic at a large hospital in Al Ain City, UAE, from January to April 2019 (n = 647). Patients’ information had been collected through Hospital Information System (HIS). The study pharmacist evaluated the data for all patients attending the internal medicine clinic on daily basis during the study period to identify eligible patients. Patients aged ≥ 21 years who met the criteria of one of the statin benefit groups requiring high- or moderate-intensity statin therapy according to the 2018 ACC/AHA guidelines were included in this study if they had no of the below exclusion criteria (number of included patients = 272, 42%).

As per 2018 ACC / AHA guideline recommendations, the following were the statins benefits groups:

1. subjects with a history of ASCVD; a high intensity statin should be considered (Class 1 recommendation).
2. subjects with a primary elevation of LDL cholesterol ≥190 mg/dL; a high intensity statin should be considered (Class 1 recommendation).
3. subjects with diabetes.
  - In adults 40 to 75 years of age with diabetes mellitus, regardless of estimated 10-year ASCVD risk, moderate-intensity statin therapy is indicated (Class 1 recommendation).
  - In adults 40 to 75 years of age with diabetes mellitus who have multiple ASCVD risk factors, it is reasonable to prescribe high intensity statin therapy (Class 2A recommendation)
  - In adults older than 75 years with diabetes mellitus, it may be reasonable to initiate statin therapy after a clinician–patient discussion of potential benefits and risks (Class 2B recommendation, Class 2A if already on statin)
  - For diabetic patients 20–39 years old, statin may be considered in case of presence of multiple diabetes specific risk enhancers such as long disease duration, albuminuria ≥ 30 mcg albumin/mg creatinine, eGFR < 60 ml/min/1.73 m2, neuropathy, and retinopathy (Class 2B recommendation)
4. subjects 40–75 years old without diabetes or ASCVD, with baseline LDL cholesterol levels of 70 to 189 mg/dL. Treatment recommendations are based on the 10-year ASCVD risk as follows:
  - Borderline risk (10-year ASCVD risk 5–7.4%): consider a moderate intensity statin if risk enhancers present (Class 2B recommendation).
  - Intermediate risk (10-year ASCVD risk 7.5–19.9%): consider moderate intensity statin (Class 1 recommendation).
  - High risk (10-year ASCVD risk ≥20%): consider high intensity statin (Class 1 recommendation).
  - In addition, the guideline recommends considering treatment with statins in individuals aged 20 to 39 years old with a family history of premature ASCVD disease and a high LDL of ≥ 160 mg/dL (Class 2B recommendation).

The main exclusion criteria were: 1) history of statin-induced rhabdomyolysis or myopathy; 2) history of allergic reaction to statins; 3) current active liver disease; 4) creatine kinase levels >3 times the upper limit of normal; 5) Any contraindications to statins use; 6. Patients for whom a lipid profile was not available or who did not have a sufficient data to classify them into statin benefit groups or enough information for calculating the ASCVD risk score at the time the study was conducted were excluded as well.

### Ethical consideration

This study was approved by the Hospital Research Ethics Committee (Ref. CR /2018/40). All methods were performed in accordance with relevant guidelines and regulations. The study was explained to patients and their consent was obtained before participation.

### Study design and data collection

**Fig 1:**
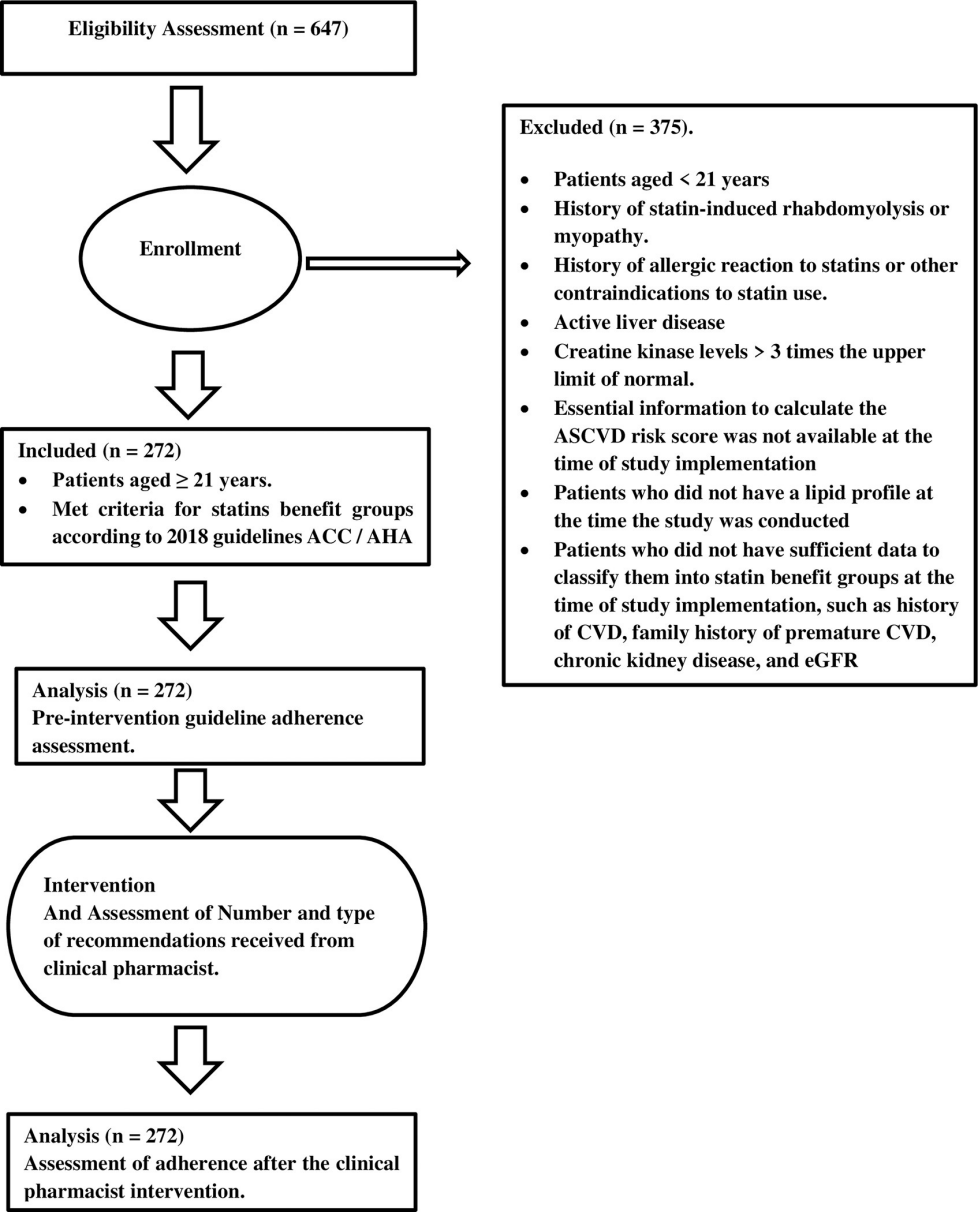
Study design flowchart.

We utilized in this study an interventional before-after design. Demographic and clinical characteristics of the study sample were extracted from the hospital information system. Data collected did not include any personal or sensitive information such as patient’s identity or medical record number. Fig 1 represents the flowchart of the study design.

### Data collection before clinical pharmacists’ intervention

Eligibility for statin therapy was evaluated based on 2018 ACC / AHA guideline recommendations as stated above. Adherence to guideline recommendations before clinical pharmacist’s intervention was measured by calculating the percentage of patients identified per each statin benefit group, the percentage of patients receiving statin therapy as per guideline recommendation, the type and intensity (moderate or high intensity) of statin therapy used, and the need for additional non-statin therapy. We calculated only adherence to class 1 and class 2A guideline’s recommendations.

### Clinical pharmacists’ interventions

The clinical pharmacists received the medication order for each patient after the patients’ appointment with the physician. The clinical pharmacists evaluated all patients’ data and the appropriateness of their medication order and recommended therapy modifications to meet the 2018 ACC/AHA cholesterol management guideline recommendations. Physicians were automatically notified with the pharmacist’s intervention in the online system and responded accordingly by agreeing, modifying, or rejecting the pharmacist’s recommendation. Physician response and treatment plan changes due to clinical pharmacist interventions were extracted from the Hospital Information System.

### Data collection after clinical pharmacists’ intervention

Adherence to guideline recommendations was measured by calculating the percentage of patients identified per each statin benefit group, the percentage of patients receiving statin therapy as per guideline recommendations, the type and intensity (moderate or high intensity) of statin therapy used, and the need for additional non-statin therapy. We calculated only adherence to class 1 and class 2A recommendations. Rejecting a recommendation that is based on a class 2B guideline recommendation was not considered as nonadherent. These class 2B recommendations are usually considered by the guideline as “may be reasonable” and “weak” where their benefit is ≥ risk.

### Study outcomes

The following outcomes were examined in this study:

1. Adherence to the 2018 ACC /AHA guideline recommendations was measured by determining the following:
  - The number and percentage of statin benefit group patients who were prescribed a statin
  - Appropriateness of statin dose (high intensity vs moderate intensity)
  - The need for additional non-statin therapy.
2. The impact of clinical pharmacist interventions on the application of guideline recommendations was determined by measuring the following:
  - The number and type of recommendations attained by the clinical pharmacist.
  - Physicians’ acceptance of recommendations
3. Comparison of differences in adherence to 2018 ACC /AHA guidelines before and after clinical pharmacist interventions.

### Statistical analysis

All data were entered and analyzed using Statistical Package for the Social Sciences (SPSS) version 22 (IBM SPSS Statistics for Windows, Version 24.0. Armonk, NY: IBM Corp.). Descriptive statistics were used to measure the frequencies and percentages. The chi-square test was used to compare adherence to guidelines before and after clinical pharmacist interventions. A p-value of 0.05 was considered statistically significant, using a 95.0% confidence interval for differences.

## Results

### Demographic and clinical characteristics of the study sample

The demographic and clinical characteristics of the study sample are shown in Table 1. The mean age of the studied patients was 52.6 ±10.5, and 71.3% (n = 194) of them were males. Majority of the patients (95.6%, n = 260) had previous illness or chronic disease. Out of these patients, 178 patients (65.4%) had hypertension, 150 patients (55.1%) had dyslipidemia, and 196 patients (72.1%) had diabetes mellitus. Nevertheless, 20 patients (7.4%) had LDL-C levels less than 70 mg/dl, 236 patients (86.8%) had LDL-C levels between 70–189 mg/dl, and 16 patients (5.9%) had LDL-C levels equal to or more than 190.

**Table 1:** Demographic and clinical characteristics of the study sample.

	Age Categories			
	21 - <40	40–75	>75	Total
Total participants	36 (13.2%)	226 (83.1%)	10 (3.7%)	272 (100.0%)
**Population characteristics**				
**Gender:**				
Male	30 (11.0%)	160 (58.8%)	4 (1.5%)	194 (71.3%)
Female	6 (2.2%)	66 (24.3%)	6 (2.2%)	78 (28.7%)
**Race:**				
White and others	28 (10.2%)	176 (64.7%)	4 (1.5%)	208 (76.5%)
Black	8 (2.9%)	50 (18.4%)	6 (2.2%)	64 (23.5%)
**Smoking**	22 (8.1%)	78 (28.7%)	2 (0.75%)	102 (37.5%)
**Hypertensive patients**	20 (7.4%)	150 (56.6%)	8 (2.9%)	178 (65.4%)
**Total cholesterol**	213±53.1	188±45.1	198±20.8	192±46.5
< 200mg/dl (< 5.2 mmol/L)	14 (5.1%)	132 (48.5%)	2 (0.75%)	148 (54.4%)
200 – 239mg/dl (5.2–6.1 mmol/L)	10 (3.7%)	58 (21.3%)	8 (2.9%)	76 (27.9%)
≥ 240mg/dl (≥ 6.2 mmol/L)	12 (4.4%)	36 (13.2%)	0 (0%)	48 (17.6%)
**HDL**	37±5.6	39±8.6	46±6.3	39±8.3
< 40mg/dl (<1.03 mmol/L)	24 (8.8%)	132 (48.5%)	0 (0%)	156 (57.4%)
≥ 40mg/dl (≥ 1.03 mmol/L)	12 (4.4%)	94 (34.6%)	10 (3.7%)	116 (42.6%)
**LDL**	128±44.2	120±37.3	135±5.1	121±38.1
< 70mg/dl (<1.81 mmol/L)	0 (0.0%)	20 (7.4%)	0 (0.0%)	20 (7.4%)
70 – 189mg/dl (1.81–4.88 mmol/L)	32 (11.8%)	194 (71.3%)	10 (3.7%)	236 (86.8%)
≥ 190mg/dl (≥ 4.9 mmol/L)	4 (1.5%)	12 (4.4%)	0 (0%)	16 (5.9%)
**Dyslipidemia and on statin**	18 (6.6%)	126 (46.3%)	6 (2.2%)	150 (55.1%)
**Diabetes and on diabetic medications**	26 (9.6%)	166 (61.1%)	4 (1.5%)	196 (72.1%)
**eGFR:**	99.3±22.8	87.1±22.3	69.6±27.8	88.3±22.9
**BMI:**	28.9±4.9	30.2±6.7	25.7±8.3	30±6.4
Mean of estimated 10 years risk of CVD*:	9.1±4.9	15.9±10.3	30.3±7.4	15.5±10.1
< 7.5%	6 (2.2%)	44 (16.2%)	0 (0%)	50 (18.4%)
≥ 7.5-<20%	22 (8%)	88 (32.4%)	4 (1.5%)	110 (40.5%)
≥20%	0 (0%)	50 (18.4%)	10 (3.7%)	60 (22.1%)
**History of CVD**	8 (2.9%)	44 (16.2%)	0 (0.0%)	52 (19.1%)
**ASCVD not at very high risk**	6 (2.2%)	16 (5.9%)	0 (0.0%)	22 (8.1%)
**Very high risk ASCVD**	2 (0.75%)	28 (10.3%)	0 (0.0%)	30 (11.0%)

*The estimated 10 years risk of CVD is measured using Pooled Cohort Risk Equation [11].

Mean risk is calculated among the participants without history of CVD.

eGFR = estimated glomerular filtration rate, HDL = high density lipoprotein, LDL = low density lipoprotein, BMI = body mass index, ASCVD = Atherosclerosis Cardiovascular Disease.

Additionally, 52 patients (19.1%) had a history of clinical ASCVD. Of these, 38 patients (14.0%) were at very high risk of recurrent CVD. Out of all participants without a history of clinical ASCVD, 50 patients (18.4%) had an estimated 10-year CVD risk less than 7.5%, 110 patients (40.5%) had an estimated 10-year CVD risk greater than or equal to 7.5% but less than 20.0%, and 60 patients (22.1%) had an estimated 10-year CVD risk equal to or greater than 20.0%.

### The adherence with the 2018 ACC/AHA guideline recommendations for the management of cholesterol in adults before clinical pharmacist interventions

Adherence with the 2018 ACC/AHA guideline recommendations for the management of cholesterol in adults is shown in Table 2. Based on the inclusion criteria, all the patients who were enrolled (100.0%, n = 272) were identified as statin benefit groups according to the 2018 ACC/AHA guideline recommendations. Of these, only 60.3% (n = 164) were initiated on statin therapy.

**Table 2:** Adherence with the 2018 ACC/AHA guideline for the management of cholesterol in adults before clinical pharmacist’s intervention.

Statin benefit groups	Total “n”	Use of statins		Statin intensity level				Addition of non-statin therapy to achieve LDL-C goals					
		Adherence	Non-adherence	Low	Moderate	High	Adherence	Ezetimibe		PCSK9 Inhibitors		Other lipid lowering agents	
								Required	Initiated	Required	Initiated	Initiated	Drug used
**History of ASCVD (Not at very high risk)**	20	14	6	0	11	3	3	7	2	0	0	0	
History of ASCVD (Very high risk⸙)	28	20	8	0	12	8	8	10	2	0	0	2	Fenofibrate 300mg
**DM / (ASCVD risk score <7.5%)**	44	26	18	4	14	8	22	10	2	0	0	0	
**DM / (ASCVD risk score ≥7.5<20%)**	74	46	28	6	23	17	17	19	4	0	0	16	Fenofibrate 145mg
**DM / (ASCVD risk score ≥20%)**	48	32	16	6	12	14	14	14	0	0	0	4	Fenofibrate 145mg
**ASCVD risk score ≥7.5<20%**	30	6	24	0	2	4	6	4	0	0	0	0	
**ASCVD risk score ≥20%**	12	8	4	0	4	4	4	4	0	0	0	0	
**LDL-C ≥ 190 mg/dl**	16	12	4	0	8	4	4	16	4	6	0	2	Gemifibrozil 600mg
**Total**	272	164 (60.3%)	108 (39.7%)	16 (9.8%)	86 (52.4%)	62 (37.8%)	78 47.6%)	84 (51.2%)	14 (8.5%)	6 (3.7%)	0 (0%)	24 (14.6%)	
**Compliance with guideline**		**60.3%**		**47.6%**				**16.7%**		**0%**			

ASCVD = Atherosclerosis Cardiovascular Disease, DM = Diabetes Mellitus, n = number of patients.

*The estimated 10 years risk of CVD is measured using Pooled Cohort Risk Equation. Risk is calculated among the participants without history of CVD.

**⸙**Very high-risk includes a history of multiple major ASCVD events or 1 major ASCVD event and multiple high-risk c

Out of those who were on statin therapy, 9.8% (n = 16) were on low intensity statin (e.g., simvastatin 10 mg and pitavastatin 1 mg), 52.4% (n = 86) were on moderate intensity statin (e.g., simvastatin 40 mg, rosuvastatin 10 mg, atorvastatin 20 mg, pitavastatin 2 mg and 4 mg) and 37.8% (n = 62) were on high intensity statin (e.g., atorvastatin 40 mg and 80 mg and rosuvastatin 20 mg and 40 mg). Adherence to the recommend level of statins intensity was identified in only 47.6% of patients (n = 78).

The addition of non-statin therapies to achieve LDL-C goals was also assessed, and ezetimibe was required for 51.2% (n = 84) of those who were on statin therapy. While it was initiated for only 8.5% (n = 14). PCSK9 inhibitors were required for 3.7% (n = 6) of those who were on statin and ezetimibe therapies. However, such treatment was not initiated in any patient.

Other lipid-lowering agents, such as fibric acid derivatives (fenofibrate 145 mg and 300 mg and gemfibrozil 600 mg), were initiated on 14.6% (n = 24) of those who were on statin therapy.

### The value of the clinical pharmacist’s interventions on applying the 2018 ACC/AHA guideline recommendations

The impact of the clinical pharmacist interventions on applying the 2018 ACC/AHA guideline recommendations is shown in Table 3. In patients with LDL-C<70 mg/dl, 18 recommendations were made, ranging from adding moderate- or high-intensity statins for those who were not initiated on statins (need additional therapy–class I and IIa recommendations as per 2018 ACC/AHA guideline definition of recommendation class), changing to moderate- or high-intensity statin agents for those who were on lower-intensity statin agents and stopping other lipid-lowering agents that may not help in achieving LDL-C goals (dose adjustment/stop unnecessary medications—class I and IIa recommendations). However, the physicians’ acceptance of the aforementioned recommendations was only 22.2%.

**Table 3:** The value of the clinical pharmacist interventions on applying the 2018 ACC/AHA guideline recommendations.

LDL group	Recommendations		Interventions		Physicians response	
	No.	Type	Type	Rational*	%	Reasons for rejecting
**Patients with LDL-C<70mg/dl**	4	Adding high intensity statin	Need additional therapy	Class I recommendation Class IIa recommendation	50%	Low LDL-C level, concerns about side effect and the additional cost
	2	Adding moderate intensity statin	Need additional therapy	Class I recommendation	100%	
	2	Change to high intensity statin	Change drug	Class IIa recommendation	0%	Low LDL-C level, concerns about side effect and the additional cost
	4	Change to high intensity statin	Dose adjustment	Class I recommendation	0%	Low LDL-C level, concerns about side effect and the additional cost
	4	Change to high intensity statin/stop fenofibrate	Dose adjustment/Unnecessary medication	Class IIa recommendation	0%	Patients can’t tolerate the higher dose
	2	Change to moderate intensity statin	Dose adjustment	Class I recommendation	0%	Low LDL-C level, concerns about side effect and the additional cost
**Total =**	**18**				**22.2%**	
**Patients with LDL-C between 70-189mg/dl**	50	Adding ezetimibe	Need additional therapy	Class I recommendation Class IIa recommendation	90%	Low LDL-C level (72), concerns about side effect and the additional cost
	8	Adding ezetimibe/stop fenofibrate	Need additional therapy/unnecessary medication	Class IIa recommendation	100%	
	82	Adding high intensity statin	Need additional therapy	Class I recommendation Class IIa recommendation	80.5%	Low LDL-C level (70–87), concerns about side effect and the additional cost
	16	Adding moderate intensity statin	Need additional therapy	Class I recommendation	87.5%	Low LDL-C level (79), concerns about side effect and the additional cost
	6	Change to high intensity statin	Change drug	Class I recommendation	100%	
	60	Change to high intensity statin	Dose adjustment	Class I recommendation Class IIa recommendation	71.7%	18.3% Patients can’t tolerate the higher dose. 10% Low LDL-C level (70–76), concerns about side effect and the additional cost
	4	Change to high intensity statin/stop fenofibrate	Change drug/ unnecessary medication	Class I recommendation	0%	Patients can’t tolerate the higher dose
	24	Change to high intensity statin/stop fenofibrate	Dose adjustment/unnecessary medication	Class I recommendation Class IIa recommendation	83.4%	Patients can’t tolerate the higher dose.
	12	Change to high intensity statin/stop fenofibrate/add ezitimibe	Dose adjustment/unnecessary medication/need additional therapy	Class I recommendation	50%	Patients can’t tolerate the higher dose.
**Total =**	**262**				**79.4%**	
**Patient with LDL-C ≥190mg/dl**	6	Adding ezetimibe/adding PCSK9/stop gemfibrozil	Need additional therapies/unnecessary medication	Class I recommendation/very high LDL-C level	66%	The decision for adding PCSK9 inhibitors has been delayed for the next follow-up
	8	Adding high intensity statin/ Adding ezetimibe	Need additional therapy	Class I recommendation/very high LDL-C level	100%	
	4	Adding PCSK9	Need additional therapy	Class I recommendation/very high LDL-C level	100%	50% not tolerating moderate or high intensity statin
	12	Change to high intensity statin/adding ezitimibe	Dose adjustment/need additional therapy	Class I recommendation/very high LDL-C level	100%	
**Total =**	**30**				**93.2%**	

* Recommendation class is as defined by the 2018 ACC/AHA management of blood cholesterol guideline

In patients with LDL-C between 70–189 mg/dl, 262 recommendations were carried out ranged from adding ezetimibe and stopping other ineffective LDL-C lowering agents for those who were in maximum tolerated dose of statin (need additional therapy/stop the unnecessary medication–class I and IIa recommendations), adding moderate or high intensity statin for those with who were not initiated on statin (need additional therapy–class I and IIa recommendations) and changing to high intensity statin dose or drug for those who were on lower intensity statin agents and stopping other ineffective LDL-C lowering agents (dose adjustment/change drug/stop the unnecessary medications—class I and IIa recommendations). The physicians’ acceptance of these recommendations was 79.4%.

In patients with LDL-C ≥190 mg/dl, 30 recommendations were submitted, ranging from adding ezetimibe, PCSK9 inhibitor and stopping gemfibrozil for those with very high LDL-C results and requiring a more than 25.0% reduction in LDL-C levels despite the use of high-intensity statins (requiring additional therapy–class I recommendation), adding high-intensity statins together with ezetimibe for those who were not on statins (requiring additional therapies–class I recommendation), adding PCSK9 inhibitors for those with high LDL-C levels, although they were on high-intensity statins together with ezetimibes (requiring additional therapy–class I recommendation) and changing to high-intensity statins and adding ezetimibe for those on moderate-intensity statins even though the LDL-C level was more than or equal to 190 mg/dl (dose adjustment/requiring additional therapy–class I recommendation). Interestingly, the physicians’ acceptance of these recommendations was 93.2%.

**Fig 2:**
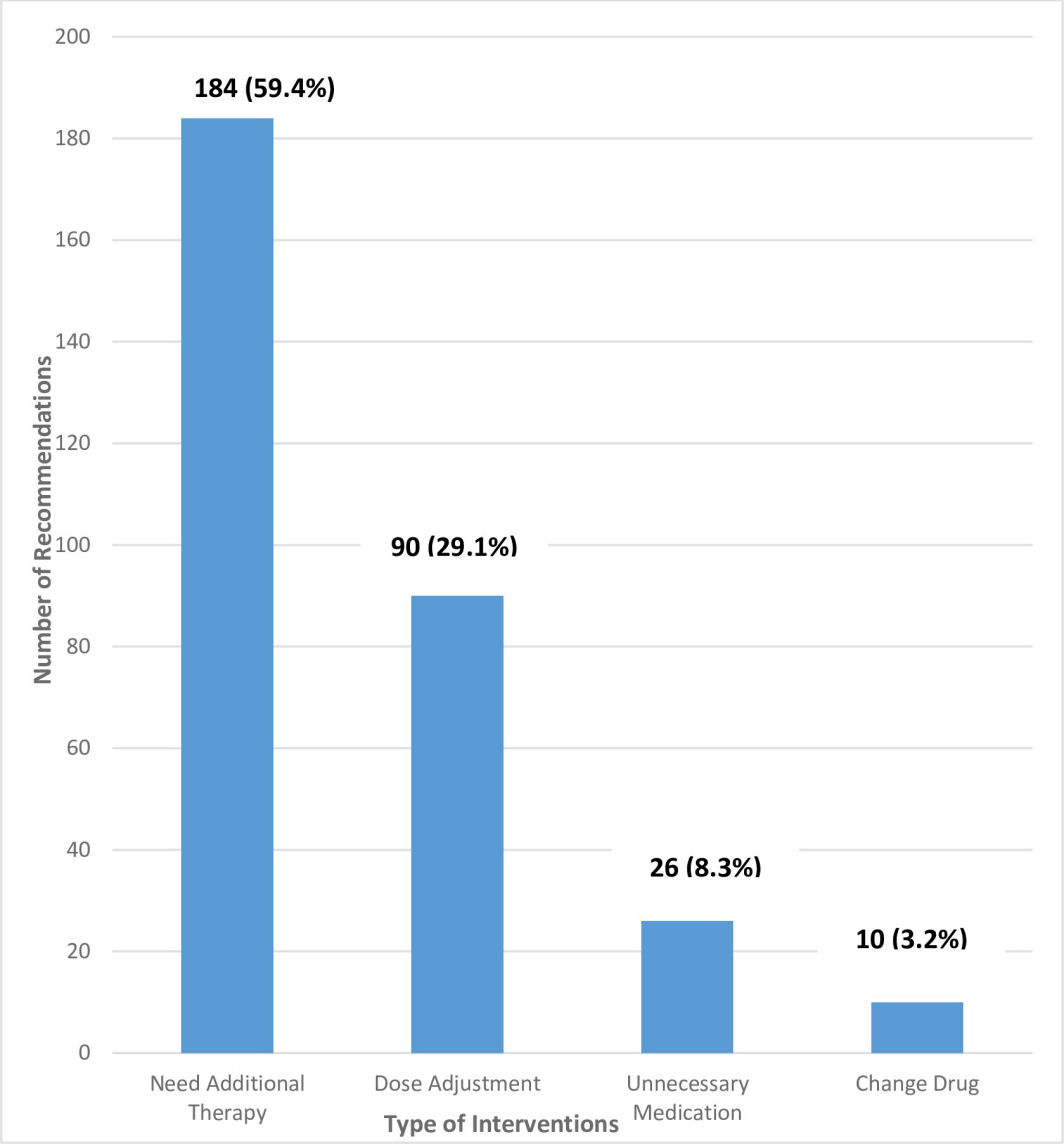
The total number of recommendations and type of interventions performed by the clinical pharmacist (n = 310).

Fig 2 summarizes the number of recommendations and type of interventions performed by the clinical pharmacist to achieve the desired outcomes.

### Adherence with the 2018 ACC/AHA guideline after clinical pharmacist’s interventions

Adherence with the 2018 ACC/AHA guideline for the management of cholesterol in adults after clinical pharmacist interventions is shown in Table 4. Accordingly, the number of patients who were initiated on statin therapy increased significantly up to 92.6% (n = 252) after the clinical pharmacist interventions were implemented (*X*^*2*^ (df = 1, n = 272) = 79.1, *p* = 0.0001).

**Table 4:** Adherence with the 2018 ACC/AHA guideline for the management of cholesterol in adults after the clinical pharmacist’s interventions.

Statin benefit groups	Total “n”	Use of statins		Statin intensity level				Adding of non-statin therapy to achieve LDL-C goals					
		Adherence	Non-adherence	Low	Moderate	High	Adherence	Ezetimibe		PCSK9 Inhibitors		Other lipid lowering agents	
								Required	Initiated	Required	Initiated	Initiated	Drug used
**History of ASCVD (Not at very high risk)**	20	18	2	0	4	14	18	7	7	0	0	0	
**History of ASCVD (Very high risk⸙)**	28	24	4	0	6	18	18	10	8	0	0	2	Fenofibrate 300mg
**DM / (ASCVD risk score <7.5%)**	44	42	2	2	20	20	40	10	10	0	0	0	
**DM / (ASCVD risk score ≥7.5<20%)**	74	66	8	2	7	57	64	19	18	0	0	2	Fenofibrate 145mg
**DM / (ASCVD risk score ≥20%)**	48	46	2	2	4	40	42	14	12	0	0	4	Fenofibrate 145mg
**ASCVD risk score ≥7.5<20%**	30	28	2	0	2	26	28	4	2	0	0	0	
**ASCVD risk score ≥20%**	12	12	0	0	0	12	12	4	4	0	0	0	
**LDL-C ≥ 190 mg/dl**	16	16	0	0	2	14	16	16	16	6	4	0	
**Total**	272	252 (92.6%)	20 (7.4%)	6 (2.4%)	45 (17.9%)	201 (79.8%)	238 (94.4%)	84 (33.3%)	77 (30.6%)	6 (2.4%)	4 (1.6%)	8 (3.2%)	
**Adherence with guideline**		**92.6%**		**94.4%**				**91.7%**		**66.7%**			

ASCVD = Atherosclerosis Cardiovascular Disease, DM = Diabetes Mellitus, n = number of patients.

*The estimated 10 years risk of CVD is measured using Pooled Cohort Risk Equation. Risk is calculated among the participants without history of CVD.

**⸙**Very high-risk includes a history of multiple major ASCVD events or 1 major

Consequently, the number of patients who were on low- or moderate-intensity statins decreased to 2.4% (n = 6) and 17.9% (n = 45), respectively. However, the number of patients who were on high-intensity statins potentially increased to 79.8% (n = 201). Based on that, adherence with the recommendations regarding the level of statin intensity used was significantly improved to 94.4% (n = 238) after the clinical pharmacist interventions (*X*^*2*^(df = 1, n = 252) = 72.5, *p* = 0.0001).

The use of ezetimibe as an add-on nonstatin therapy was encouraged and effectively added to the treatment plan to achieve LDL-C goals. The number of patients who were initiated ezetimibe increased significantly to 91.7% (n = 77) after the clinical pharmacist interventions (*X*^*2*^ (df = 1, n = 84) = 95, *p* < 0.0001).

Interestingly, for those who were on statin and ezetimibe therapies and required PCSK9 inhibitors to achieve LDL-C goals, adherence with the recommendations was effectively improved to 66.7% (n = 4); (*X*^*2*^ (df = 1, n = 6) = 6, *p* = 0.014). The use of other lipid-lowering agents, such as fibrates, was markedly reduced to 3.2% (n = 8) for those who were on statin therapy after the clinical pharmacist interventions (*X*^*2*^(df = 1, n = 208) = 19.2, *p* < 0.0001).

**Fig 3:**
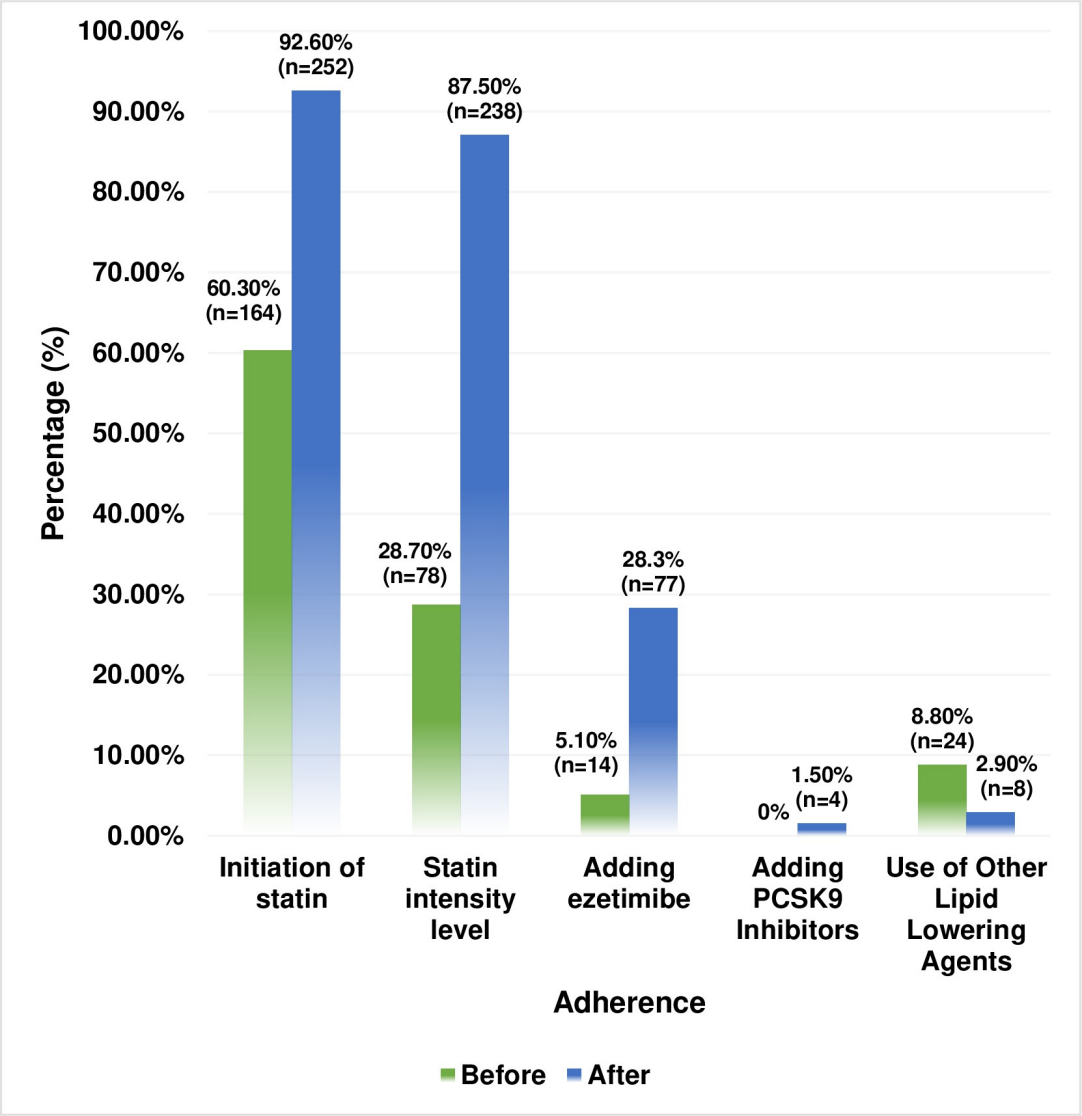
Comparison of adherence with the 2018 ACC/AHA guideline recommendations before and after clinical pharmacists’ interventions (n = 272).

Fig 3 shows comparison of adherence with the 2018 ACC/AHA guideline recommendations for the management of cholesterol before and after clinical pharmacist interventions regarding the initiation of statins, the proper use of moderate- or high-intensity statins, evidence-based addition of ezetimibe and PCSK9 inhibitors and minimization of other lipid-lowering agent abuse.

## Discussion

Based on this study, adherence with the 2018 ACC/AHA guideline recommendation for the management of cholesterol in adult patients before clinical pharmacist interventions was 60.3% for the initiation of statins therapy and 47.6% for adherence to proper intensity statin therapy. Accordingly, the initiation of statins, particularly high-intensity statins, is prescribed to far fewer patients than recommended. Consequently, the use of non-statin therapies such as ezetimibe and PCSK9 inhibitors was nearly diminished, taking into consideration that several studies highlighted the importance of pharmacist intervention on cholesterol risk management and revealed the treatment gap between research evidence and clinical practice [12–15].

According to our findings, the clinical pharmacist plays a crucial role in the management of cholesterol levels by recommending new therapies, adjusting or increasing drug doses and stopping or changing medications. Furthermore, systematic reviews and meta-analyses of randomized trials conducted by Machado et al. and Santschi et al. emphasized the importance of pharmaceutical care interventions in the management of CVDs [16, 17]. Pharmacist interventions achieved greater reductions in systolic and diastolic blood pressure (BP), total cholesterol (TC), and LDL-C and in the risk of smoking compared with the usual care group [16, 18, 19]. Nevertheless, various clinical trials have illustrated great benefits of statin use, such as pleiotropic effects, which could be beneficial for the treatment and management of several comorbidities [20–22].

In this study, adherence with the 2018 ACC/AHA guideline to achieve the required LDL-C goals was significantly improved after clinical pharmacist interventions and the implementation of the appropriate recommendations. Consistently, Bozovich et al. 2000 and Tahaineh et al. 2011 showed significant improvement in achieving LDL-C goals when clinical pharmacists managed lipid clinics or through clinical pharmacy services under the supervision of cardiologists [23, 24]. The same was achieved by Tsuyuki RT et al. (2016) [25].

In the current study, physicians’ acceptance of the clinical pharmacist’s recommendation according to the guidelines was variable based on patients’ LDL-C levels. For instance, physicians” acceptance of clinical pharmacist interventions was high among patients with LDL-C ≥70 mg/dl. Subsequently, this resulted in greater improvement of LDL-C levels and improvement in health outcomes. Likewise, recent studies reported that primary healthcare physicians significantly relied on clinical pharmacists in assessing and improving patients’ adherence to their medications as well as in educating and counseling the patients to avoid clinical malpractice and achieve better health outcomes [26, 27].

Several studies presented the major explanations for statin refractoriness reported by healthcare practitioners, and patients were concerned about adverse events [12, 28–33]. Rosenson, R. S 2016 stated that evaluation of potential adverse events requires validated tools to differentiate between statin-associated adverse events versus nonspecific complaints. Additionally, treatment options for statin-intolerant patients include the use of different statins, often at a lower dose or frequency. To lower LDL cholesterol, lower doses of statins may be combined with ezetimibe or bile acid sequestrants [34]. Newer treatment options for patients with statin-associated muscle symptoms may include proprotein convertase subtilisin kexin 9 (PCSK9) inhibitors [12].

There are many reasons that contribute to non-adherence with the guidelines or rejecting the pharmacists’ recommendations such as the availability of specific drugs and patient’s reluctant for treatment initiation or dose escalation. The results of the current study indicated that many physicians are reluctant to prescribe high intensity statins due the worry about side effects and myopathy. In some cases, physicians stop or change the dose of statins when patients’ report intolerance of such medications or due to the high cost. Another reason for rejecting the pharmacists’ recommendations in this study was low bassline LDL-C level for some patients despite the patient being categorized as a statin benefit group.

In clinical trials, statin-associated adverse events showed no differences between participants assigned to statins or placebo [12, 28]. However, it is important to know that these trials select patients with better tolerability and lower risk for myopathy based on their ages, absence of musculoskeletal complaints, normal renal function and less concomitant medications that may alter the pharmacokinetic pathways [12, 29]. One of the solutions to overcome the problem is to switch to the fully human monoclonal antibodies proprotein convertase subtilisin/kexin type 9 (PCSK9) inhibitors (alirocumab and evolocumab) that caused fewer muscle symptoms based on clinical trials and were no more often than when ezetimibe was used [30–32]. However, the cost of such treatment is still one of the main barriers.

### Limitations

This study is an interventional before after design. This design is usually used in circumstances where it is not possible to use a control group for ethical or practical issues. Although this design is suitable for the current study, the lack of control group makes this design prone to bias and many confounders. Therefore, the outcome can instead be related to any changes that occurred around the same time as the intervention. Although the clinical pharmacists and physicians who treated the patients in the studied clinics remained the same during the study period, other unknown confounders could have occurred. Another limitation is that it is not an easy task to initiate statin therapy for those are low or intermediate risk for ASCVD. It involves looking for wide range of risk-enhancing factors which could favor the initiation of statin therapy for the low or intermediate risk group, for example the presence of premature CVD in the family. These risk-enhancing factors can be missed during patient assessment or during data extraction. on the other hand, the patient with low or intermediate ASCVD risk is allowed to option for not taking statins for primary ASCVD prevention. Therefore, it is difficult to precisely judge physicians for adherence to guideline for initiating lipid-lowering agents. In addition, the estimate ASCVD risk is more imprecise in some patients when cholesterol levels were used after treatment.

## Conclusions

The clinical pharmacist has a key role in improving the management of blood cholesterol by recommending therapies, adjusting doses and stopping or changing medications. Furthermore, adherence with the latest updated guideline recommendations to achieve the desired treatment goals was notably enhanced after the clinical pharmacist interventions and the implementation of the appropriate recommendations. This study illustrates how collaboration between physicians and clinical pharmacists can be crucial strategy to improve patients’ treatment and hence, achieve better health outcomes among patients suffering from dyslipidemia.

## Supporting information

S1 Data (XLSX)
